# Nutritional and immunization status of under-five children of India and Bangladesh

**DOI:** 10.1186/s40795-021-00484-6

**Published:** 2021-12-02

**Authors:** Sreeparna Banerjee, SubirBiswas, Shimul Roy, Manoranjan Pal, Md. Golam Hossain, Premananda Bharati

**Affiliations:** 1 Department of Anthropology, West Bengal State University, Berunanpukuria, PO-Malikapur, Barasat, West Bengal 700126 India; 2 Department of Anthropology, Vidyasagar University, Midnapore, West Bengal 721102 India; 3 Economic Research Unit, Indian Statistical Institute, 203 BT Road, Kolkata, West Bengal 700 108 India; 4 Department of Statistics, University of Rajshahi, Rajshahi, 6205 Bangladesh; 5 Biological Anthropology, Indian Statistical Institute, 203 BT Road, Kolkata, West Bengal 700 108 India

**Keywords:** BCG, DPT, Polio, Measles, Underweight, Stunting

## Abstract

**Background:**

The nutritional and immunization status of children can play an important role in determining their future health status of a particular country. The aim of the present study is to investigate the nutritional and immunization status of under-five children in India and Bangladesh, and to find the difference in the status between these two countries.

**Methods:**

We have used the National Family Health Survey data, 2015–2016 of India and Bangladesh Demographic Health Survey, 2017–2018 datasets. The sample sizes are 222,418, among them 8759 and 8759 children for India and Bangladesh respectively. The nutritional status of under-five children is measured by standard anthropometric indicators of height-for-age (HAZ) and weight-for-age (WAZ). Regarding child immunization status, only BCG, DPT, polio and measles vaccinations are considered for the present study. Multiple binary logistic model has been used for analysing the data.

**Results:**

This study reveals that the prevalence of stunting and underweight of under-five children in India are higher than Bangladeshi children. Secondary and higher educated mothers are more likely of having normal HAZ and WAZ children than up to primary educated mothers for both countries. Chances of having normal HAZ and WAZ are higher among non-poor category for both countries. The present study also shows that immunization status of Bangladeshi children is better than Indian children except measles. Religion of mother also shows influence on immunization status of children in India whereas Bangladesh shows no significant results regarding religion. Mother’s educational attainment and wealth index show influence on immunization status among children for both countries.

**Conclusions:**

The study concludes that a remarkable number of under-five children are suffering from under nutrition for both countries, however Bangladeshi children have better nutritional and immunization status compared to Indian children. Higher wealth index, better educational attainment and lower unemployment of Bangladeshi mothers may be the causes for better nutritional and immunization status of children. Mother’s socio-economic factors have significant impact on determining the child’s health status. Our findings can help to government of Indian and Bangladesh for taking health policy to improve under-five children nutritional and immunization status.

## Background

Malnutrition is the silent killer that affects human development and economy of any country. Children of today are citizens of tomorrow, so children’s nutritional status plays an important role in determining the future of our country and should be prioritized. Child height and weight are considered as important indicators of population health and human capital [1, 2]. United Nations Children’s Fund pointed out that globally 165 million children under the age-5 years are found to be stunted (low- height-for -age), 101 million children are underweight (low weight for age) and 52 million children are wasted (weight for height) [3]. According to WHO 2002 estimated that in developing countries, 60% of the 10.9 million deaths that occurs annually among children aged less than 5 years are association with under nutrition, approximately 25% of deaths in under five occur in India alone which is association with improper feeding practices [4]. Indian children are shorter than elsewhere in the developing world at similar level of economic development [1]. According to the recent report of Global Hunger Index 2020, revealed that India ranking 94th out of 107 countries with a score 27.2, and has the third highest score in Asia [5]. As of 2015–2016, more than a fifth (21%) of children in India suffered from wasting (low weight for height) and progression regarding stunting (low height for age), down by 20% since 2005, the rate still stands at a staggering 38.4%. Underweight rate is reduced by 16% since 2005 but even that progress leaves India with a relatively high rate of 35.7% [6].

India being a principal producer and exporter of vaccines, but still, it is home to one-third of the world’s under- five children with no immunization [7, 8]. The South Asian countries was home to 1.8 of the 6 million babies who died within twenty- eight days of birth in 2015 [9]. The research depicts that the number of deaths from measles fell from more than 535,000 in 2000 to approximately 139,000 in 2010. Approximately half of these deaths occurred in India attributed to lower vaccination rates [10]. Approximately, three million children die annually due to vaccine preventable diseases in developing countries [11]. More than 85% of children from Nepal and Bangladesh were fully immunized compared to 43.6% from India [9]. Child immunization status is as important as child nutritional status. Many studies revealed that child immunization and nutritional status depends on parent’s socio-economic status [12–14]. Various research revealed that Gender of the child, family income and parental education have significant impact on immunization status of children [15–22].

By 2050, 25 million more children than today will suffer from malnourishment [23]. Bangladesh ranks 77th among 107 countries with a score 20.4 [5], and India, Nigeria and Pakistan are considered as the home to almost half (47.2%) of all stunted children. Largest numbers of children who are stunted are from India (46.6 million), Nigeria (13.9) and Pakistan (10.7 million). Prevalence of wasting is also extremely high in these three counties; India (25.5 million) and Nigeria (3.4 million) also Indonesia (3.3 million) [24]. In developing nations, 98% of world’s undernourished people and two third of developing countries’ unprivileged people stay in Bangladesh, China, Democratic Republic of Congo, Ethiopia, India, Indonesia, Pakistan and among them India and China together account for 40% [25].

In Bangladesh, death rate among children under the age 5 declined from 133 per 1000 live births in 1993 to 46 per 1000 in 2014 [26]. Recently Bangladesh has experienced a vigorous economic growth, with the 20th highest average increase (6.3%) in gross domestic product of any country globally since 2008 and its GDP is projected to be the 41st largest by 2020 (International Monetary Fund 2016) [27]. This prolonged period of economic growth has connection to the improvements in child health [28].

Child growth and development are highly influenced by living standard, socio-economic, and demographic factors, cultural and climatic factors that can vary across the nations [29, 30]. A large number of studies reported the impact of early childhood malnutrition in developing countries [31–37]. Many studies explored that more childhood deaths occurred in low and middle-income countries [38]. An important factor for preventing diseases and death in childhood is immunization, which plays a crucial role in dropping down the child mortality rates globally [39, 40]. The Bangladesh Demographic and Health survey 2004 depicts that higher level of maternal education are associated with lower risk of underweight among children [41]. Paternal education also has significant impact in determining the status of child health [14, 42]. Many research revealed the reasons for the poor immunization status of children in developing countries [8, 21, 43, 44]. Some of the research revealed that lack of knowledge concerning the vaccine preventable diseases, the reason of immunization, age at which the child should start and complete immunization was associated with the lower immunization status among children in developing countries [22, 44].

Vaccines can develop the immune system and also improve overall health and nutrition. A multi-country level study reported that incomplete vaccinations had cusses for stunting underweight and wasting among under -five children in different countries including Bangladesh [45].

### Key research questions

(i) How many under-five children in India and Bangladesh are suffering from under nutrition?
(ii) What is immunization status of under-five children in India and Bangladesh?
(iii) Is there any difference and/or similarities in nutritional and immunization status among under-five children between two countries?
(iv) Has mother’s socio-economic background impact on child’s nutritional status and immunization status for both countries?

The aim of the present study is to investigate nutritional and immunization status among under-five children of India and Bangladesh. In addition, we want to look the difference in nutritional and immunization status among under-five children between India and Bangladesh.

## Methods

### Study area and population

The data of the National Family Health Survey (NFHS-4 of 2015–2016) of India and Bangladesh Demographic and Health Survey (BDHS-2017-2018) of Bangladesh are used for this study. It provides information on various socio-economic and demographic variables of women aged 15–49 years and their children aged 0–59 months, however, in the present study we considered children aged 1–59 months, as the sample size of 0 months age is very small in both the countries.

### Data collection procedures

NFHS-4 fieldwork is carried out in two phases (20 January 2015 to 4 December 2016). The sample size is 628,900 households. However, the Interviews are successfully completed only in 601,509 households. Among 723,875 ever-married women aged 15–49 years, 699,686 women are interviewed. All the states of India are included in the study and all the Union Territories except Delhi are excluded from the study. In the present study, we consider only those women aged 15–49 years, who conceived baby within last 5 years. The respondents provide information regarding socio economic and demographic factors both for women and men. All the selected women and men are members of the sampled households or visitors who stayed in the sampled households the night before the survey. NFHS-4 canvasses four types of questionnaires namely the household questionnaire, the women’s questionnaire, the men’s questionnaire and biomarker questionnaire. As this study is related to women’s and her child, the analysis is restricted to the woman’s questionnaire only. For children under 5 years, information on age is collected by birth certificate. Height and weight are measured for children aged 1–59 months. The Seca 874 digital scales used to measure the weight of the children [6].

In Bangladesh (BDHS-217-2018) field work is carried out in five phases, each about 4 weeks in duration. Data collection starts on 24 October 2017 and ends on 15 March 2018. Here the sample size is 20,160, which is much less than the sample size taken in India. However, the interviews are successfully completed in 19,457 households. Among 20,376 ever-married women aged 15–49 years, 20,127 women are interviewed. BDHS-2017-2018 canvasses five types of questionnaires: household questionnaire, the women’s questionnaire (ever-married women aged 15–49 years), biomarker questionnaire, verbal autopsy questionnaire to collect data on causes of death among children under age 5 and the community questionnaire. In the present study, we restrict our study only to women questionnaire. In the present study the sample size consists of 231177children, of which 222,418 are from India and 8759 are from Bangladesh. The present study mainly focuses on the health status of children with respect to socio-economic and demographic factors of mother. It is a comparative as well as ex-post-facto research.

### Measurement of nutritional and immunization status

Child malnutrition is measured by standard anthropometric indicators of height-for-age (HAZ) and weight-for-age (WAZ). These two anthropometric measurements are commonly used as proxy for child malnutrition [46]. Stunting is defined as low height for age while underweight is low weight for age. A child is considered as stunted if the child’s HAZ score falls below minus two times the SD below the median of the WHO reference population, where SD means standard deviation. Similarly, A child is considered as underweight, if the child’s WAZ score falls below minus two times the SD below the median of the WHO reference population [47, 48]. Among the child immunization variables, only BCG, DPT, polio and measles vaccinations are considered.

The details explanation of survey design, sampling technique, data collection procedure, measuring system, validity of the measurement and quality control have been found elsewhere in IIPS [6] for NFHS-4 and NIPORT-2020 [49] for BDHS 2017–2018.

### Outcome variable

The outcome (dependent) variable of this study is child’s nutritional and immunization status. Nutritional status is measured by (i) stunting (‘Yes’ is coded as 1; ‘No’ as 0) and (ii) underweight (‘Yes’ is coded as 1; ‘No’ as 0). Similarly, the immunization status of BCG, DPT, polio, and measles are measured by the binary variable taking values 0 for ‘No’ and 1 ‘Yes’, where ‘Yes’ means the vaccine has been taken and ‘No’ means the vaccine has not been taken.

### Independent variable

The independent variables taken for the study are the six socio-economic and demographic factors. Those are educational attainment of mother (up to primary level, at secondary level and higher than secondary level), residential pattern (rural and urban), mother’s occupation (not working, professional, sales, agriculture work, other work), religion (Hindu, Muslim, and other religious group, which includes Christians, Jains, Buddhists etc.), wealth index (poor and non-poor) and number of children (1 or 2; more than 2 children). The above variables have been selected on the basis of a previous study [50] and availability in NFHS-4 and BDHS 2017–2018 datasets.

### Statistical analysis

Frequency distribution is used to determine the prevalence of nutritional and immunization status. Multiple binary logistic regression model is utilized to find associated factors of children nutritional and immunization status. Value of *p* < 0.05 is considered as statistically significant in the analysis. All statistical analyses are performed using SPSS (IBM Version 21).

## Results

A total number of 231,177 under-five children are considered as sample. The mean ages of children are 30.02 and 28.81 months with age range 1–59 months in both India and Bangladesh. It is noted that the prevalence of stunting and underweight of under-five children are respectively 39.7% and 34.8% in India and respectively 31.8% and 22.6% in Bangladesh. The rates of stunting and underweight among Indian children are higher than that of Bangladesh children. The highest percentages of stunting and underweight are observed among children with the lowest educational group of mothers, and the rates of stunting and underweight of children decrease with increase of mothers’ education level in both the countries. In India, the highest percentages of stunting and underweight children are observed among mothers engaged in agricultural labour and other work. The lowest percentage of stunted and underweight children is observed among families with mother’s being engaged in professional work in both the countries. In Bangladesh, the highest percentages of stunted and underweight children are observed among mothers engaged in services and agricultural work.

The study also reveals that, the higher percentages of stunted and underweight children are in rural areas compared to children in urban areas in both India and Bangladesh. Higher percentages of stunted and underweight children are found among households of poor wealth index category in both the countries. In India, the percentage of underweight children is higher in Hindu religious group, whereas in Bangladesh, the higher percentage of underweight children is observed among other religion households. The lowest percentage of stunted children is observed among children belonging to other religious group in India, whereas in Bangladesh, the lowest percentage of stunted children is observed among Hindus. In India, the lowest percentage of underweight children is found in children belonging to other religious group, whereas in Bangladesh, the lowest percent of underweight children belongs to Hindu religion. The study also highlights that higher percentage of stunted and underweight children are found among women having more than 2 children in both India and Bangladesh (Table 1).

**Table 1:** Frequency distribution of nutritional status of under-five children in India and Bangladesh by different socio-economic and demographic factors

Socio-economic and demographic variables	India		Bangladesh		India		Bangladesh	
	Stunted N (%)	Normal N (%)	Stunted N (%)	Normal N (%)	Underweight N (%)	Normal N (%)	Underweight N (%)	Normal N (%)
**Education**								
Up to Primary	48,170 (48.8)	50,627 (51.2)	1148 (40.8)	1663 (59.2)	43,702 (43.3)	57,321 (56.7)	860 (29.8)	2028 (70.2)
Secondary educated	33,102 (34.0)	64,136 (66.0)	1116 (30.4)	2554 (69.6)	29,509 (29.5)	70,484 (70.5)	800 (21.1)	2986 (78.9)
Higher sec and above educated	4301 (22.1)	15,182 (77.9)	197 (15.5)	1072 (84.5)	3778 (18.7)	16,438 (81.3)	146 (11.0)	1176 (89.0)
**Residence**								
Rural	69,152 (41.8)	96,291 (58.2)	1750 (34.2)	3372 (65.8)	62,120 (36.6)	107,581 (63.4)	1270 (24.1)	4003 (75.9)
Urban	16,421 (32.8)	33,654 (67.2)	711 (27.1)	1917 (72.9)	14,869 (28.9)	36,662 (71.1)	536 (19.7)	2187 (80.3)
**Occupation**								
Not working	10,819 (38.0)	17,621 (62.0)	1308 (29.6)	3113 (70.4)	9709 (33.2)	19,502 (66.8)	980 (21.5)	3584 (78.5)
Professional	162 (24.7)	493 (75.3)	25 (15.5)	136 (84.5)	128 (18.5)	563 (81.5)	23 (13.6)	146 (86.4)
Sales	150 (39.8)	227 (60.2)	18 (23.1)	60 (76.9)	115 (29.6)	273 (70.4)	12 (15.4)	66 (84.6)
Agriculture	2350 (46.7)	2684 (53.3)	820 (36.1)	1450 (63.9)	2119 (41.2)	3021 (58.8)	572 (24.4)	1768 (75.6)
Service	330 (39.2)	511 (60.8)	103 (39.0)	161 (61.0)	272 (31.2)	601 (68.8)	81 (30.0)	189 (70.0)
Other	768 (43.6)	994 (56.4)	187 (33.8)	366 (66.2)	750 (41.9)	1038 (58.1)	138 (24.1)	434 (75.9)
**Religion**								
Hindu	63,130 (40.3)	93,451 (59.7)	192 (31.1)	426 (68.9)	59,395 (37.0)	101,134 (63.0)	141 (22.4)	488 (77.6)
Muslim	13,849 (41.2)	19,801 (58.8)	2254 (31.8)	4831 (68.2)	11,679 (33.8)	22,906 (66.2)	1654 (22.6)	5665 (77.4)
Others	8594 (34.0)	16,693 (66.0)	15 (31.9)	32 (68.1)	5915 (22.6)	20,203 (77.4)	11 (22.9)	37 (77.1)
**Wealth Index**								
Poor	64,087 (44.7)	79,238 (55.3)	1334 (40.3)	1977 (59.7)	57,698 (39.3)	89,285 (60.7)	963 (28.4)	2430 (71.6)
Non-poor	21,486 (29.8)	50,707 (70.2)	1127 (25.4)	3312 (74.6)	19,291 (26.0)	54,958 (74.0)	843 (18.3)	3760 (81.7)
**Number of children**								
1 to 2	45,480 (35.5)	82,732 (64.5)	1630 (29.5)	3900 (70.5)	42,501 (32.3)	89,261 (67.7)	633 (27.6)	1664 (72.4)
More than 2	37,477 (46.3)	43,547 (53.7)	831 (37.4)	1389 (62.6)	32,145 (38.7)	50,869 (61.3)	1173 (20.6)	4526 (79.4)

The study shows the frequency distribution of receiving different vaccines for improving immunization status of children with respect to mother’s socio-economic and demographic factors. It is observed that the highest percentage of children is immunized with BCG vaccine, DPT vaccination, polio and measles vaccination belonging to higher educational group of mothers in both the countries except in few cases. The lowest percentage of children taking vaccination belongs to mothers with the lowest educational status in India as well as in Bangladesh. The study also depicts that higher percentages of children in urban areas are immunized with BCG, DPT, polio and measles vaccination than children residing in rural areas in both the countries. The highest percentages of children immunized with DPT, BCG, Measles and polio belong to mothers engaged in professional work in India. While in Bangladesh, the highest percent of children immunized with BCG belongs to mother’s engaged in professional group. The highest percentages of children immunized with DPT, polio and measles belong to mothers engaged in other work. In India, the study also reveals that the highest percentage of children who are not immunized with BCG, DPT and polio vaccination are the children of mothers engaged in agricultural work. The highest percentage of not immunized with measles are found among children of not working mothers. The higher percentages of Hindu children received BCG, DPT, polio and measles vaccination compared to Muslim and other religious groups. While in Bangladesh, the highest percentage of children immunized with BCG belongs to mothers engaged in sales work. The highest percentages of children who are not taking BCG vaccination are found among not working mothers. The highest percentages of children immunized with DPT, Polio and measles vaccination belong to mothers engaged in other work. The highest percentage of children who are not immunized with DPT, polio and measles belongs to not working group of mothers. The higher percentage of children belonging to non-poor wealth index get immunized with BCG, DPT, polio and measles than children belong to poor wealth index group in both the countries. The study also points out that the highest percentage of children belonging to other religion is immunized with BCG and measles. The highest percentage of Hindu children is immunized with DPT and polio vaccination. In India and Bangladesh, women having 1 or 2 children show higher percentage of taking vaccinations than women having more than 2 children (Table 2).

**Table 2:** Frequency distribution of receiving vaccines in India and Bangladesh by socio-economic and demographic groups

Socio-eco and demographic factors	Child immunization in India							
	BCG, Yes N(%)		DPT, Yes N(%)		Polio, Yes N(%)		Measles, Yes N(%)	
**India**								
**Education**	No	Yes	No	Yes	No	Yes	No	Yes
Up to Primary	15,699 (15.5)	85,304 (84.5)	36,348 (36.1)	64,242 (63.4)	41,193 (40.8)	59,871 (59.2)	35,309 (35.2)	64,939 (64.8)
Secondary educated	8192 (8.2)	92,253 (91.8)	24,636 (24.6)	75,515 (75.4)	31,280 (31.1)	69,139 (68.9)	27,590 (27.6)	72,366 (72.4)
Higher sec and above educated	957 (4.7)	19,483 (95.3)	3972 (19.5)	16,409 (80.5)	5555 (27.2)	14,881 (72.8)	4903 (24.1)	15,476 (75.9)
**Residence**								
Rural	20,276 (11.9)	149,744 (88.1)	51,669 (30.5)	117,738 (69.5)	61,359 (36.1)	108,693 (63.9)	53,364 (31.6)	115,602 (68.4)
Urban	4572 (8.8)	47,296 (91.2)	13,287 (25.7)	38,428 (74.3)	16,669 (32.1)	35,198 (67.9)	14,438 (28.0)	37,179 (72.0)
**Occupation**								
Not working	3029 (10.3)	26,295 (89.7)	8191 (28.0)	21,060 (72.0)	9938 (33.9)	19,394 (66.1)	9038 (31.0)	20,156 (69.0)
Prof	40 (5.7)	663 (94.3)	133 (19.0)	569 (81.0)	185 (26.3)	518 (73.7)	153 (21.9)	546 (78.1)
Sales	47 (12.1)	342 (87.9)	111 (28.5)	279 (71.5)	117 (30.0)	273 (70.0)	107 (27.6)	281 (72.4)
Agri	671 (13.1)	4466 (86.9)	1647 (32.2)	3472 (67.8)	1895 (36.9)	3247 (63.1)	1525 (29.8)	3590 (70.2)
Service	85 (9.7)	794 (90.3)	205 (23.4)	671 (76.6)	257 (29.2)	622 (70.8)	228 (26.1)	647 (73.9)
manual	167 (9.3)	1621 (90.7)	424 (23.8)	1360 (76.2)	516 (28.8)	1275 (71.2)	443 (24.9)	1333 (75.1)
**Religion**								
Hindu	14,602 (9.1)	146,399 (90.9)	43,834 (27.3)	116,617 (72.7)	55,054 (34.2)	105,888 (65.8)	45,538 (28.4)	114,609 (71.6)
Muslim	5441 (15.7)	29,219 (84.3)	12,288 (35.6)	22,230 (64.4)	13,546 (39.1)	21,119 (60.9)	12,612 (36.6)	21,817 (63.4)
Others	4805 (18.3)	21,422 (81.7)	8834 (33.8)	17,319 (66.2)	9428 (35.8)	16,884 (64.2)	9652 (37.1)	16,355 (62.9)
**Wealth index**								
Poor	19,737 (13.4)	127,426 (86.6)	48,030 (32.8)	98,555 (67.2)	55,711 (37.8)	91,493 (62.2)	48,584 (33.2)	97,581 (66.8)
Non-poor	5111 (6.8)	69,614 (93.2)	16,926 (22.7)	57,611 (77.3)	22,317 (29.9)	52,398 (70.1)	19,218 (25.8)	55,200 (74.2)
**Number of children**								
1 to 2	10,127 (7.7)	122,185 (92.3)	32,670 (24.8)	99,227 (75.2)	42,008 (31.8)	90,294 (68.2)	35,942 (27.3)	95,750 (72.7)
More than 2	13,507 (16.3)	69,586 (83.7)	29,884 (36.1)	52,911 (63.9)	33,130 (39.9)	49,999 (60.1)	29,571 (35.8)	52,927 (64.2)
**Bangladesh**								
**Education**								
Up to Primary	175 (10.0)	1568 (90.0)	427 (24.5)	1315 (75.5)	445 (25.5)	1298 (74.5)	1026 (59.0)	713 (41.0)
Secondary educated	144 (5.9)	2304 (94.1)	422 (17.2)	2025 (82.8)	445 (18.2)	1999 (81.8)	1264 (51.8)	1178 (48.2)
Higher sec and above educated	51 (5.5)	874 (94.5)	159 (17.2)	767 (82.8)	163 (17.6)	763 (82.4)	489 (52.8)	437 (47.2)
**Residence**								
Rural	252 (7.5)	3112 (92.5)	678 (20.2)	2686 (79.8)	708 (21.1)	2653 (78.9)	1818 (54.1)	1541 (45.9)
Urban	118 (6.7)	1634 (93.3)	330 (18.8)	1421 (81.2)	345 (19.7)	1407 (80.3)	961 (55.0)	787 (45.0)
**Occupation**								
Not working	273 (8.8)	2836 (91.2)	685 (22.0)	2423 (78.0)	716 (23.0)	2391 (77.0)	1751 (56.4)	1352 (43.6)
Prof	5 (4.5)	106 (95.5)	22 (19.8)	89 (80.2)	22 (19.8)	89 (80.2)	59 (53.2)	52 (46.8)
Sales	1 (2.3)	43 (97.7)	6 (13.6)	38 (86.4)	7 (15.9)	37 (84.1)	23 (52.3)	21 (47.7)
Agriculture	64 (4.7)	1305 (95.3)	219 (16.0)	1150 (84.0)	228 (16.7)	1141 (83.3)	710 (51.9)	658 (48.1)
Service	11 (7.5)	135 (92.5)	31 (21.2)	115 (78.8)	33 (22.6)	113 (77.4)	80 (55.6)	64 (44.4)
Other work	16 (4.8)	318 (95.2)	45 (13.5)	289 (86.5)	47 (14.1)	286 (85.9)	155 (46.4)	179 (53.6)
**Religion**								
Hindu	23 (5.9)	366 (94.1)	54 (13.9)	335 (86.1)	56 (14.4)	332 (85.6)	184 (47.3)	205 (52.7)
Muslim	347 (7.4)	4353 (92.6)	949 (20.2)	3750 (79.8)	991 (21.2)	3707 (78.9)	2583 (55.1)	2108 (44.9)
Others	__	27 (100.0)	5 (18.5)	22 (81.5)	6 (22.2)	21 (77.8)	12 (44.4)	15 (55.6)
**Wealth index**								
Poor	166 (7.7)	1982 (92.3)	463 (21.6)	1685 (78.4)	478 (22.3)	1669 (77.7)	1220 (56.9)	924 (43.1)
Non-poor	204 (6.9)	2764 (93.1)	545 (18.4)	2422 (81.6)	575 (19.4)	2391 (80.6)	1559 (52.6)	1404 (47.4)
**Number of children**								
1 to 2	240 (6.4)	3513 (93.6)	708 (18.9)	3045 (81.1)	743 (19.8)	3008 (80.2)	2008 (53.6)	1737 (46.4)
More than 2	130 (9.5)	1233 (90.5)	300 (22.0)	1062 (81.1)	310 (22.8)	1052 (77.2)	771 (56.6)	591 (43.4)

The findings of the Table 3 confirm that mother’s educational attainment has statistically significant influence on nutritional status of children in both countries (*p* < 0.01). Mother’s occupational status also plays an important role in determining the nutritional status of children, mothers engaged in agricultural work are more likely to have stunted children in India than not working mother and the result is significant (*p* < 0.01). Chance of getting underweight is higher among children of mother’s engaged in agricultural work and it is highly significant in case of India (*p* < 0.01). The chances of being stunted or underweight among children of mothers engaged in service are the highest in Bangladesh followed by group “Other workers” and the result is significant (*p* < 0.05) for mothers engaged in service. The chance of being stunted is higher among children of mother with more than 2 children compared to children with mother having 1 or 2 children for both countries (*p* < 0.01). The study revealed that the chance of stunting is more among children resides in rural area than children in urban area and the result is significant (*p* < 0.01), in India while no significant association are observed between residential pattern and underweight status of children in Bangladesh. While in Bangladesh, no significant association are noted between nutritional status and residential pattern, but the study revealed that children reside in urban area have better nutritional status than children in rural area. Children belonging to Hindu religion have more chance to become stunted and underweight than children belonging to Muslim and other religious groups in India (*p* < 0.01). Bangladesh shows no significant association between nutritional status and religious group. The study shows that children belong to better wealth index group have better nutritional status than children in poor wealth index group in both the countries (*p* < 0.01) (Table 3). Hosmer and Lemeshow test has been used in this study to find the goodness of fit of our selected model. The *p*-value of the test is greater than 0.05, Hosmer and Lemeshow test demonstrated that our selected model was good fitted for both countries data regarding children nutritional status.

**Table 3:** Effect of socio-economic and demographic factors on nutritional status of children in India and Bangladesh using binary logistic regression

	Stunting		Underweight	
**Socio-economic and demo variable**	**India**	**Bangladesh**	**India**	**Bangladesh**
	AOR, 95% CI of AOR (Lower-Upper)	AOR, 95% CI of AOR (Lower-Upper)	AOR, 95% CI of AOR (Lower-Upper)	AOR, 95% CI of AOR (Lower-Upper)
**Educational attainment** (Up to primary level®)				
Secondary education	1.518 (1.446–1.594)**	1.379 (1.235–1.540)**	1.582 (1.505–1.662)**	1.395 (1.238–1.572)**
Higher sec. and above	2.458 (2.232–2.708)**	2.781 (2.3001–3.362)**	2.744 (2.479–3.037)**	2.708 (2.187–3.352)**
**Occupation** (Not working®)				
Profession	1.002 (0.829–1.211)	1.039 (0.661–1.634)	1.094 (0.892–1.342)	0.819 (0.512–1.312)
Sales	0.824 (0.662–1.026)	1.404 (0.817–2.413)	0.943 (0.748–1.190)	1.518 (0.810–2.843)
Agriculture	0.907 (0.850–0.967)**	0.955 (0.852–1.071)	0.914 (0.857–0.976)**	1.066 (0.940–1.208)
Service	0.887 (0.764–1.029)	0.767 (0.590–0.997)*	1.017 (0.871–1.187)	0.750 (0.568–0.989)*
Other work	0.920 (0.832–1.017)	0.842 (0.694–1.021)	1.193 (1.135–1.253)**	0.880 (0.714–1.083)
**Number of children** (More than 2®)				
1 to 2	1.265 (1.205–1.327)**	1.111 (0.994–1.241)	0.996 (0.940–1.056)	1.180 (1.048–1.329)**
**Residence** (Rural®)				
Urban	1.086 (1.026–1.149)**	1.118 (0.997–1.253)	0.996 (0.940–1.056)	1.086 (0.958–1.231)
**Religion** (Hindu®)				
Muslim	1.123 (1.056–1.194)**	1.013 (0.843–1.217)	1.383 (1.299–1.473)**	1.055 (0.864–1.289)
Others	1.379 (1.282–1.483)**	0.950 (0.494–1.825)	2.069 (1.911–2.239)**	0.978 (0.480–1.994)
**Wealth Index** (Poor®)				
Non-poor	1.399 (1.326–1.476)**	1.512 (1.351–1.691)**	2.069 (1.911–2.239)**	1.404 (1.242–1.586)**

® Reference category, *AOR* Adjusted odds ratio, *CI* Confidence interval, **p < 0.01, *p < 0.05; Source: Data Extracted from NFHS 4, 2015–2016. Dependent variable = nutritional status (0 = stunted/underweight, 1 = normal)

The present study noted that the relation of socio-economic and demographic factors on child immunization status in India and Bangladesh. The chance of having better immunization status is higher among children of mother with higher educational attainment in both the countries and the result is significant (*p* < 0.01) except measles vaccination (in Bangladesh). The study also noted that chance of being immunized with DPT increases among children of mother engaged in profession, service and other work than not working women (*p* < 0.01). Study also depicts that chance of being immunized with measles vaccination among children increases among working mother than not working mother in case of India (*p* < 0.05). The study shows that polio and BCG vaccination have significant (*p* < 0.01) relation with mother’s occupational status except in few cases. Highly significant association are noted between total number of children and immunization status of the children in India (*p* < 0.01) while in Bangladesh, similar result is found but no significant relation is noted for stunting of children. India and Bangladesh show no significant association between immunization status of children and residential pattern of children. The study also shows that Hindu children have higher chance for immunization than Muslim and other religion children in India and Bangladesh (*p* < 0.01). Children in non-poor wealth index group are more likely to be immunized than poor children and it is significant (*p* < 0.01) except BCG and polio in Bangladesh. While children immunization shows significant association with wealth index of mother, and it is significant (*p* < 0.01) (Table 4). We observed that Hosmer and Lemeshow test demonstrated that our selected model was well fitted for children immunization status of both countries.

**Table 4:** Effect of socio-economic and demographic factors on immunization status of children in India and Bangladesh using binary logistic regression

Socio-eco and demographic factors	India				Bangladesh			
	DPT	Polio	Measles	BCG	DPT	Polio	Measles	BCG
	AOR	AOR	AOR	AOR	AOR	AOR	AOR	AOR
**Educational attainment (Up to primary educated®)**								
Secondary education	1.49**	1.39**	1.28**	1.73**	1.49**	1.49**	1.27**	1.72**
Higher sec and above	1.68**	1.52**	1.32**	2.26**	1.50**	1.57**	1.19	1.79**
**Occupation (Not working®)**								
Profession	1.26*	1.16	1.41**	1.23	0.89	0.93	1.05	1.43
Sales	1.01	1.19	1.37**	1.02	1.84	1.61	1.22	4.32
Agriculture	1.02	1.04	1.24**	1.05	1.71**	1.71**	1.29**	2.33**
Service	1.29**	1.25**	1.32**	1.20	1.16	1.14	1.12	1.35
Other work	1.38**	1.36**	1.43**	1.29**	1.79**	1.79**	1.50**	1.88*
**Number of child (More than 2®)**								
1 to 2	1.39**	1.29**	1.26**	1.69**	1.12	1.09	1.07	1.43
**Residence (Rural®)**								
Urban	1.02	1.04	0.99	0.96	1.08	1.10	0.92	1.17
**Religion (Hindu®)**								
Muslim	0.84**	0.99	0.83**	0.79**	0.68*	0.68**	0.76*	0.90
Others	0.78**	0.95	0.71**	0.51**	0.70	0.58	1.16	1.02
**Wealth Index (Poor®)**								
Non-poor	1.28**	1.15**	1.20**	1.45**	1.19*	1.15	1.22**	1.03

*®* Reference category, *AOR* Adjusted odds ratio, **p < 0.01, *p < 0.05; Source: Data Extracted from NFHS 4, 2015–2016. Dependent variable = Immunization status of children (0 = No immunization, 1 = Yes)

## Discussion

Since there are overwhelmingly large collection of studies showing that parents’ education, especially mother’s level of education is positively related with the status of health and immunisation of children, it is needless to compare our results with results of others, because we also have found similar results. Household standard of living in terms of wealth, income or per capita expenditures also has uncontroversial relationships with children’s health and immunisation status. It is also known that the households living in urban areas are better equipped and informed and hence health and immunisation status of urban children are better than those of rural children.

The present study noted that prevalence of stunting and underweight among under-5 years children are more in Indian children than Bangladesh. The study also revealed that mother’s socio-economic condition effects child nutritional and immunization status in both the country. Higher percentage of mother is found to be not working in India (76.6%) than in Bangladesh (57.1%). Most of the studies made so far on this line are by taking only two groups; ‘working’ and ‘not working’. So, it is difficult to compare with our results. For example, in one of the studies reveals that in Eritrea, did not get much difference between the immunization status of children of working and non-working mothers. They found that 82.8% children of working mothers were completely immunized as against 83.4% children of non-working mothers. The difference was not significant enough [51]. Using data of a Government General Hospital in India, [52] also didn’t find significant difference between the immunization status of children of unemployed and employed mothers, though in this case the percentage of complete immunization among children of employed mothers were slightly higher. Preschool children mainly depend on breastfeeding and may be protected by mother’s immune system at birth. Breastfeed protects children from infectious diseases and affect the nutritional status of the children [53]. Some study depicts that with increasing age of child, child need complementary food in addition to breastfeeding. Inadequate complementary food also might be a reason of increasing malnutrition in developing countries [54].

So far as parental occupations are concerned, the results are mixed type. We have in this paper taken a number of occupational status of parents, like ‘not working’, ‘profession’, ‘sales’, ‘agriculture’, ‘service’ and ‘other work’ and we have found significant effect of these occupations on the status of health and immunisation of children when compared it with the category ‘not working’. The present study reveals that the children of mothers engaged in agriculture and sales are more likely to be underweight or stunted than the children of not working women in India. However, the result is not statistically significant. The situation is almost opposite in Bangladesh, where the children have less chance in these two categories. The chances of children being stunted (0.767 times) and underweight (0.750 times) are higher among children of mothers engaged in service than of not working mothers. So far as children’s health status is concerned, the association between health statuses of children with employment status of parents was found to be significant. In fact, child’s poor health status is found to be associated with reduced maternal and paternal employment [55]**.** Many studies on childhood vaccination depicts that parental education have a significant influence on child immunization [56–59]. It may be due to better knowledge regarding immunisation schedules of children than non-educated parents [60]. The chance of getting immunization among children is higher among mothers who are engaged in professional work, sales, agricultural work, services, and other work than not working mother and the result is significant except for few cases.

The present study revealed that mother’s educational level has a significant impact on child health and the result is significant in both the countries. Similarly, in other studies, we observed that the children of educated mothers were fully immunised compared to non-educated mothers [57, 58, 61]. The present study reveals that the chance of getting immunization of children increases as mother’s educational level increases. Better immunization among children is observed among mother’s who are engaged in service and the result is significant except BCG vaccination. The chance of being immunized among children increases whose mothers are engaged in other work and it is significant at 1% level. Parental literacy has a significant impact on children immunization status [62]. The present study depicts that in India, the chance of being normal (not underweight) is higher among children of secondary educated mothers (odd ratio = 1.582) and highest among children of higher secondary or above educated mothers (odd ratio 2.744) than primary educated mother. Similar result is found in case of stunting. Thus, women’s literacy level plays an important role in reducing child’s malnourishment as found by many other studies [33, 63, 64]. Many studies depict a significant association of mother’s education on child’s stunting [65–67]. In Bangladesh, the chance of getting stunted children is lower among secondary educated mothers (1.379 times) and higher and above educational group (2.781 times) mothers than mothers educated up to primary level. Underweight children are less likely to be observed among secondary educated mothers (1.395 times) and higher and above educated group of mothers (2.708 times) than mothers educated up to primary level. Children with parents with no education were more likely to be underweight than those from parents with secondary or higher level of education [68]. The study also revealed that women with better education level, have better immunization status of their children and it is significant at 1% level.

Women having one or two children are (1.265 times) more likely to have normal (not- stunted) children than women having 2 or more children, and the result is statistically significant. The study also highlighted that religion also influence child health status. A lower immunization status was found in Muslim families than Hindu. Religion also shows significant relation with child immunization status except polio vaccination. The study also reveals that the chance of getting BCG vaccination among children is higher among mothers having 1 or 2 children than 2 or more children. Regarding wealth index, percentage of household with non-poor wealth index in Bangladesh (58%) is found to be higher than in India (33.6%). The study also reveals that the chance of getting immunization becomes less among children with higher household size and the result is significant. Much research revealed that in Bangladesh, the immunization status between the richest and poorest wealth index was statistically significant [68, 69]. Again, household size of the family appeared be an important factor of fully immunised children. Children who belong to larger family size were more likely to be unimmunised. Many similar studies were conducted in Indonesia, Greece, and Angola. They reported that children from larger family size were less likely to be fully immunised [70, 71].

The present study reveals that women having better wealth index have a higher chance to have normal children (not stunted) (1.399 times) than women belong to poor wealth index. Similar result is observed in case of underweight. The chance of being stunted is less among non-poor children (1.512 times) than poor children and it is significant. The chance of underweight is higher among non-poor children (1.404 times) than children belong to poor wealth index group.

Urban children have better nutritional status than rural children, but no significant association is observed between residential pattern and child nutritional status. The chances of getting DPT (1.277 times), polio (1.152 times) and measles (1.198 times) vaccination are higher among non-poor children than children belong to poor wealth Index group. Vaccination of children is associated with wealth index, distance from health care facility, mother’s age, mother’s education, etc. [14]. Parental education and household wealth appear to be the two most important predictor of child malnutrition [72, 73].

## Conclusions

The study finds that socio-economic and demographic factors play important role in increasing likelihood of children’s nutritional status and immunization status. Nutritional status and immunization status is better among children of Bangladesh compared to India. Stunting (38.5%) and underweight (34.6%) among Indian under-five children are higher than Bangladeshi children (stunting, 28.1%; underweight, 20.6%). Mother’s educational attainment and wealth index play the most significant roles in determining children’s nutritional and immunization status. Bangladesh needs to keep up the good performance and India needs to seriously focus on its child nutritional programs. Higher wealth index, better educational attainment and lower unemployment of Bangladeshi mothers may be the causes of better performances of Bangladesh so far as better nutritional status of children in Bangladesh is concerned. India and Bangladesh governments should improve health policy to increase nutritional and immunization status of under-five children, we hope our findings can help for this purpose.

## Data Availability

The BDHS-2017-2018 and the NFHS-4 datasets are freely available at https://dhsprogram.com/data/dataset/Bangladesh_Standard-DHS_2017.cfm?flag=0 and https://dhsprogram.com/data/dataset/India_Standard-DHS_2015.cfm?flag=0 respectively.

## Abbreviations

NFHS-4: National Family Health Survey
BDHS: Bangladesh Demography Health Survey
HAZ: Height for age
WAZ: Weight for age
AOR: Adjusted odds ratio
SD: Standard deviations
WHO: World health organization
UNICEF: United Nations Children’s Fund
SPSS: Statistical package for the social science
